# Second-line treatment for renal cell cancer

**DOI:** 10.1038/bjc.2011.611

**Published:** 2012-02-14

**Authors:** G Di Lorenzo, S De Placido, C Buonerba

**Affiliations:** 1 Dipartimento di Endocrinologia ed Oncologia Clinica e Molecolare, Università Federico II, Napoli, Italy

In metastatic renal cell carcinoma (mRCC), the transition to the targeted therapy era has made available a variety of effective new agents, including vascular endothelial growth factor tyrosine kinase receptor (VEGF-TKR) inhibitors, such as sunitinib, pazopanib, sorafenib; anti-VEGF monoclonal antibody bevacizumab; and mTOR (mammalian target of rapamycin) inhibitors, everolimus and temsirolimus. A statistically significant improvement in progression-free survival (PFS) was obtained with each of these six biological drugs in well-conducted phase III trials, in comparison with either interferon alone (in the first-line setting) or placebo (in the second-line setting). As a consequence, all of these drugs have received regulatory approval in specific subsets of mRCC patients both in the US and in Europe (Gore and Larkin, 2011). Recently, VEGF-TKR inhibitor axitinib has proved to be a valuable second-line option in patients who have received targeted agents (Rini *et al*, 2011).

With the rapidly growing number of biological drugs active in mRCC, the need to establish the optimal sequence of administration is becoming more and more compelling.

In the first-line setting, no comparative phase III trial of targeted agents has been carried out (Gore and Larkin, 2011), although a number of indirect comparisons and economic evaluations involving sunitinib and bevacizumab are available. Sunitinib was associated to a prolonged PFS with respect to bevacizumab plus interferon in an indirect comparison analysis (Thompson Coon *et al*, 2009), but bevacizumab was more cost-effective than sunitinib in one study (Ravasio *et al*, 2011), although this finding was not replicated in another study (Benedict *et al*, 2011). Pazopanib has also recently emerged as an effective first-line treatment option (Gore and Larkin, 2011). Although cross-trial comparison is always challenging, pazopanib may be better tolerated than sunitinib, with a minor incidence of cardiovascular, endocrinologic and dermatologic adverse events, and it could be employed in selected patients (e.g., those with cardiovascular co-morbidities) (Di Lorenzo *et al*, 2011). Results from an ongoing head-to-head comparative phase III trial of first-line pazopanib *vs* sunitinib are eagerly awaited (COMPARZ trial, NCT 00720941).

In the second-line setting, the only FDA- and EMA-approved drug after progression on VEGF-TK inhibitors is currently everolimus, which yielded a statistically meaningful PFS advantage (4.9 *vs* 1.9 months), with no difference in overall survival (OS), with respect to placebo, in a large phase III trial (Motzer *et al*, 2010). Progression-free survival obtained with everolimus was numerically superior to that obtained with second-line sorafenib in 52 patients with sunitinib-refractory mRCC by Di Lorenzo *et al* (2009). Importantly, although all of the patients enrolled in the Di Lorenzo trial had received a single agent for metastatic disease, 79% of patients in the RECORD-1 trial had received more than one medication for systemic disease at enrollement, including sunitinib, sorafenib or interferon. An indirect comparison analysis was performed by matching patients enrolled in these two trials for histology, prior treatment, and MSKCC risk score showed that everolimus was associated with a statistically significant improved median PFS (40.8 *vs* 17.7 weeks) and improved median OS (78 *vs* 32 weeks) with respect to sorafenib (Di Lorenzo *et al*, 2011). Interestingly, these data seem to be confirmed by another retrospective review of 108 mRCC patients treated with either the TKR inhibitor –TKR inhibitor sequence (46 patients), or with the TKI-EV sequence (62 patients). Although there was no difference in PFS, the estimated OS was statistically meaningfully longer for the rTKI-EV group (43 mo; 95% CI, 33.9–52.1) than for the rTKI-rTKI group (29 mo; 95% CI, 18.6–39.5; *p*=0.03), but was only numerically longer at multivariable analysis (Busch *et al*, 2011). All of these data taken together support the notion that everolimus may be a better second-line choice than sorafenib. Furthermore, everolimus has been shown to preserve its efficacy in individuals who were intolerant to first-line TKR inhibitor, and may be particularly suitable for these patients (Bracarda *et al*, 2011). Treatment in the second-line setting has been further complicated by the recently published results obtained in the AXIS trial in patients pre-treated with one prior line of therapy, including interferon, bevacizumab or sunitinib, and randomized to either axitinib or sorafenib (Rini *et al*, 2011). Median PFS was 6.7 months with axitinib compared with 4.7 months with sorafenib (hazard ratio 0.665; 95% CI 0.544–0.812) in the whole sample population, but the PFS advantage was smaller in patients previously treated with sunitinib (4.8 *vs* 3.4 months; hazard ratio 0.741; 95% CI 0.573–0.958), which underlines the critical importance of previous therapy with targeted agents. Axitinib is likely to be soon approved for second-line use in mRCC and become an alternative to everolimus.

The third-line setting remains largely unexplored, and all of the available evidence is provided by small, retrospective studies. In a recent case study published on this *Journal*, Grünwald *et al* (2011) presented retrospective data regarding treatment of 40 patients with mRCC, who received VEGF-directed targeted agents before and after the use of everolimus. Sunitinib, sorafenib and combination of bevacizumab and interferon were administered to 75%, 23% and 3% of patients, respectively, as a first-line treatment. All patients of the study sample received either second- or third-line everolimus and were subsequently treated with a VEGF-directed targeted drug, that is to say sunitinib, sorafenib, bevacizumab/interferon and dovitinib in 48%, 20%, 8% and 25% of patients, respectively. Collected data seemed to provide some evidence in favor of the efficacy of retreatment with a VEGF-directed targeted drug following everolimus, considering that median PFS with either a third- or forth-line VEGF-directed targeted agent was overall 5.5 months. Differently from the study by Grunwald *et al*, we conducted a retrospective study of patients with mRCC, who had undergone the exact sequence sunitinib-mTOR inhibitor-sorafenib (Di Lorenzo *et al*, 2010). Importantly, of 150 medical records considered, a substantial proportion (about 25%) received third-line treatment with sorafenib, which was associated to a median PFS of 4 months and an OS of 7 months.

A possible biological explanation for this finding, which can be considered satisfactory in the third-line setting, is based on the existence of two mTOR complexes: mTORC1, which is formed by mTOR binding to the FK-binding protein and is targeted by everolimus, and mTORC2, which is not inhibited by everolimus and can be responsible for a compensatory increase of the hypoxia-inducible factor (Rini, 2010). Activation of the VEGF pathway can be consequently targeted by third-line use of TKr inhibitors. Resistance to VEGF-directed targeted agents has been simplified into two main mechanisms: one involving the potentiation of the VEGF axis and the other based on the activation of alternative pathways and growth factors (Powles *et al*, 2011). Interestingly, the former model can be employed to explain the second-line activity of axitinib, a potent and selective (unlike sunitinib, pazopanib and sorafenib) inhibitor of VEGF-r. In fact, VEGFr may still be activated during VEGF-directed therapies via a number of mechanisms, such as increased VEGF production or receptor gene mutation, and the use of a more potent VEGFr inhibitor can be clinically meaningful. On the other hand, a number of separate pathways have been identified as possibly mediating acquired resistance to VEGF-directed biological agents. Activation of these pathways can stimulate angiogenesis both directly and indirectly via a number of proteins, such as fibroblast growth factor, ephrin and angiopoietin family proteins, interleukin-8 and PlGF. In this regard, it must be noted that activity of combination of synergism of bevacizumab and interferon may be explained by the bFGF-inhibiting activity of interferon. Furthermore, preliminary evidence suggests efficacy of an angiopoietin-2 inhibitor, AMG386, which is currently being investigated in two ongoing phase II trials in combination with either sunitinib or sorafenib (Rini *et al*, 2011).

In conclusion, in patients at good- and intermediate-prognosis with clear cell mRCC sunitinib presently appears the best first-line choice, with pazopanib as a valuable alternative in selected populations. In this regard, it must be kept in mind that most of the available evidence in the second-line setting was obtained in sunitinib-pretreated patients, so efficacy data in patients treated with first-line pazopanib are lacking. Both axitinib and everolimus may be employed interchangeably as second- and third-line therapies. Everolimus may be used after axitinib, in view of the fact that the RECORD-1 trial also enrolled patients who had received two targeted therapies, and that the number of prior agents employed was not predictive of PFS. On the other hand, axitinib effectiveness may be enhanced after use of everolimus, for the reasons explained before. Temsirolimus is the only recommended treatment for patients with non-clear cell histology and those at poor prognosis, for whom a TKI-based second-line treatment is a reasonable option.

Additional trials are eagerly awaited, in order to provide evidence regarding the best choice and sequence of administration of targeted agents in mRCC.
